# A codon-optimized green fluorescent protein for live cell imaging in *Zymoseptoria tritici*^☆^

**DOI:** 10.1016/j.fgb.2015.03.022

**Published:** 2015-06

**Authors:** S. Kilaru, M. Schuster, D. Studholme, D. Soanes, C. Lin, N.J. Talbot, G. Steinberg

**Affiliations:** a Biosciences, University of Exeter, Exeter EX4 4QD, UK; b Mathematics, University of Exeter, Exeter EX4 3QF, UK

**Keywords:** FPs, fluorescent proteins, eGFP, enhanced green fluorescent protein, AcGFP, *Aequorea coerulescens* green fluorescent protein, ZtGFP, *Z. tritici* codon-optimized green fluorescent protein, GFP, green fluorescent protein, Val, valine, Arg, arginine, Ser, serine, Cys, cysteine, Ile, isoleucine, Tyr, tyrosine, Leu, leucine, His, histidine, *tub2*, α tubulin, *sdi1*, succinate dehydrogenase 1, RB and LB, right and left border, dpi, days post infection, ROI, region of interest, *n*, sample size, Green fluorescent protein, Protein localization, Wheat pathogenic fungi, *Septoria tritici* blotch, *Mycosphaerella graminicola*

## Abstract

Fluorescent proteins (FPs) are powerful tools to investigate intracellular dynamics and protein localization. Cytoplasmic expression of FPs in fungal pathogens allows greater insight into invasion strategies and the host-pathogen interaction. Detection of their fluorescent signal depends on the right combination of microscopic setup and signal brightness. Slow rates of photo-bleaching are pivotal for *in vivo* observation of FPs over longer periods of time. Here, we test green-fluorescent proteins, including *Aequorea coerulescens* GFP (AcGFP), enhanced GFP (eGFP) from *Aequorea victoria* and a novel *Zymoseptoria tritici* codon-optimized eGFP (ZtGFP), for their usage in conventional and laser-enhanced epi-fluorescence, and confocal laser-scanning microscopy. We show that eGFP, expressed cytoplasmically in *Z. tritici*, is significantly brighter and more photo-stable than AcGFP. The codon-optimized ZtGFP performed even better than eGFP, showing significantly slower bleaching and a 20–30% further increase in signal intensity. Heterologous expression of all GFP variants did not affect pathogenicity of *Z. tritici*. Our data establish ZtGFP as the GFP of choice to investigate intracellular protein dynamics in *Z. tritici*, but also infection stages of this wheat pathogen inside host tissue.

## Highlights

• We generated a *Z. tritici* codon-optimized gene for green fluorescent protein (ZtGFP).
• In epi-fluorescence and confocal microscopy, ZtGFP is brighter and more stable than eGFP.
• We provide 3 vectors that carry AcGFP, eGFP and ZtGFP for yeast recombination-based cloning.
• The vectors carry carboxin resistance for targeted integration.
• The carboxin resistance conveying vectors integrate as single copies into the *sdi1* locus.

## 1 Introduction

Live cell imaging has greatly facilitated our understanding of the invasion strategies and cell biology of pathogenic fungi. For example, the establishment of fluorescent proteins in the rice blast fungus *Magnaporthe oryzae* demonstrated that autophagy is pivotal for infection (Kershaw and Talbot, 2009) and septins scaffold penetration peg organization during early invasion of the host plant (Dagdas et al., 2012). In the corn smut fungus *Ustilago maydis*, green-fluorescent protein, fused to a putative receptor involved in membrane fusions, revealed the existence of highly mobile endosomes in fungi (Wedlich-Soldner et al., 2000). Interestingly, *in planta* observation of these organelles and fluorescent effector proteins revealed that endosome motility is crucial for effector secretion and, consequently, for virulence in *U. maydis* (Bielska et al., 2014). Thus, visualization of the dynamic behavior of fluorescent fusion proteins allows unique insight into the ways in which pathogenic fungi invade their hosts.

A fluorescent protein for live cell imaging must meet certain criteria. The protein needs to be bright enough to allow signal perception over the auto-fluorescent background. It should also be photo-stable to allow long-term observation. Finally, the protein needs to be non-toxic when expressed in cells. The green fluorescent protein (GFP) meets these requirements. This FP was first identified in the jellyfish *Aequorea victoria*, where it works in concert with the calcium-binding blue fluorescent protein aequorin (Shimomura et al., 1962). The gene encoding GFP was cloned in 1992 (Prasher et al., 1992), and the break-through for live cell imaging of GFP came when the FP was stably expressed in prokaryotic and eukaryotic cells, highlighting its potential as a reporter of protein localization and expression (Chalfie et al., 1994; Inouye and Tsuji, 1994). Since then, GFP has been used in numerous organisms in a very wide range of applications, including the study of protein localization and cellular dynamics, protein expression analysis, protein–protein interactions studies and biosensors (e.g. Garamszegi et al., 1997; Kahana and Silver, 2001; Voss et al., 2013). In filamentous fungi, GFP from *A. victoria* was first used in the corn smut fungus *U. maydis* (Spellig et al., 1996) and *Aureobasidium pullulans* (Vanden Wymelenberg et al., 1997) to visualize these inside the plant. Subsequently, GFP was used in a broad range of fungi (overview in Lorang et al., 2001); including *Mycosphaerella graminicola* (=*Zymoseptoria tritici*, Rohel et al., 2001). However, the GFP-variants used showed relatively low brightness, due to low expression and slow protein folding, and relatively poor photo-stability. These limitations were overcome in two ways. Firstly, individual amino acid residues were mutated, and secondly, the gene for *gfp* was codon-optimized to increase expression levels. An example of such a synthetically optimized protein is “enhanced” GFP (eGFP), which is improved in fluorescent brightness due to two point mutations (S65T and F64L). In addition, the codon-optimized gene carries 190 silent mutations to adapt for codon usage in humans, which increased mRNA translation rates (Haas et al., 1996; Yang et al., 1996). In fungi, codon-optimized eGFP was used in *Botrytis cinerea.* Adapting the gene for GFP to the codon usage in this fungus led to an increase in fluorescent brightness by ∼12-fold (Leroch et al., 2011). In this report, we adapt the gene for eGFP to the codon usage in *Z. tritici*. We express this ZtGFP, eGFP and a GFP from the jellyfish *Aequorea coerulescens* (AcGFP), previously used to visualize hydrophobins in *Fusarium verticillioides* (Fuchs et al., 2004), in yeast-like cells of *Z. tritici.* We then compare photo-bleaching and the brightness of the three GFPs in conventional and laser-based epi-fluorescence and confocal laser scanning microscopy. Our results show that ZtGFP, expressed from codon-optimized *egfp*, performs better than either eGFP or AcGFP for analysis of *Z. tritici*.

## 2 Materials and methods

### 2.1 Bacterial and fungal strains and growth conditions

*Escherichia coli* strain DH5α was used for the maintenance of plasmids. *Agrobacterium tumefaciens* strain EHA105 (Hood et al., 1993) was used for maintenance of plasmids and subsequently for *A. tumefaciens*-mediated transformation of *Z. tritici. E. coli* and *A. tumefaciens* were grown in DYT media (tryptone, 16 g/l; yeast extract, 10 g/l; NaCl, 5 g/l; with 20 g/l agar added for preparing the plates) at 37 °C and 28 °C respectively. The fully sequenced *Z. tritici* wild-type isolate IPO323 was used as recipient strain for the genetic transformation experiments. Cells were maintained as glycerol stocks (NSY glycerol; nutrient broth, 8 g/l; yeast extract, 1 g/l; sucrose, 5 g/l; glycerol, 700 ml/l), and cultures were grown on YPD agar (yeast extract, 10 g/l; peptone, 20 g/l; glucose, 20 g/l; agar, 20 g/l) at 18 °C for 4–5 days.

### 2.2 Molecular cloning

Plasmid pJ244-ZtGFP carries codon-optimized *ztgfp* gene and was obtained from DNA 2.0 (Menlo Park, CA, USA). All other vectors in this study were generated by *in vivo* recombination in the yeast *Saccharomyces cerevisiae* DS94 (MATα, *ura3-52*, *trp1-1*, *leu2-3*, *his3-111*, and *lys2-801* (Tang et al., 1996) following published procedures (Raymond et al., 1999; Kilaru and Steinberg, 2015). For all the recombination events, the fragments were amplified with 30 bp homologous sequences to the upstream and downstream of the fragments to be cloned (see Table 1 for primer details). PCR reactions and other molecular techniques followed standard protocols (Sambrook and Russell, 2001). The DNA fragments of interest were excised from the agarose gel and purified by using silica glass suspension as described previously (Boyle and Lew, 1995). Plasmid DNA was isolated from the positive yeast colonies as described previously (Hoffman and Winston, 1987). All restriction enzymes and reagents were obtained from New England Biolabs Inc (NEB, Herts, UK).

### 2.3 Construction of vectors pCAcGFP and pCZtGFP

Vector pCeGFP was described in Kilaru et al. (2015a). Vector pCAcGFP contains *acgfp* under the control of *Z. tritici tub2* promoter for integration in to the *sdi1* locus by using carboxin as selection agent. A 12,704 bp fragment of pCeGFPTub2 (digested with *Zra*I; Schuster et al., 2015), 1149 bp *tub2* promoter (amplified with SK-Sep-14 and SK-Sep-15; Table 1) and 720 bp *acgfp* (amplified with SK-Sep-79 and SK-Sep-80; Table 1) were recombined in *S. cerevisiae* to obtain the vector PCAcGFP (AcGFP was kindly provided by Syngenta, Basel, Switzerland). Vector pCZtGFP contains *ztgfp*, amplified from pJ244-ZtGFP (see above), under the control of *Z. tritici tub2* promoter for integration in to the *sdi1* locus by using carboxin as selection agent. A 12704 bp fragment of pCeGFP-Tub2 (digested with *Zra*I), 1149 bp *tub2* promoter (amplified with SK-Sep-14 and SK-Sep-15; Table 1) 720 bp *ztgfp* (amplified with SK-Sep-101 and SK-Sep-102; Table 1) were recombined in *S. cerevisiae* to obtain the vector pCZtGFP. Further details on vector construction and yeast recombination-based cloning are provided in Kilaru and Steinberg (2015).

### *2.4* *Z. tritici* transformation and molecular analysis of transformants

The vectors pCAcGFP, pCeGFP and pCZtGFP were transformed into *A. tumefaciens* strain EHA105 by heat shock method (Holsters et al., 1978) and *A. tumefaciens*-mediated transformation *of Z. tritici* was performed as described previously by Zwiers and De Waard (2001) with the slight modifications. Further details on this method are provided in Kilaru et al. (2015a). To confirm the integration of vector in to the *sdi1* locus and also to determine the copy number, Southern blot hybridizations were performed by using standard procedures (Sambrook and Russell, 2001). Approximately 3 μg of genomic DNA of IPO323 and transformants obtained with vectors pCAcGFP, pCeGFP and pCZtGFP were digested with *Bgl*II and separated on a 1.0% agarose gel and capillary transferred to a Hybond-N^+^ membrane (GE healthcare, Little Chalfont, United Kingdom). 1014 bp *sdi1* probe (3′ end of the *sdi1* gene and *sdi1* terminator) was generated by using DIG labeling PCR mix (Life Science Technologies, Paisley, UK) with primers SK-Sep-10 and SK-Sep-13 (Table 1). Hybridizations were performed at 62 °C for overnight autoradiographs were developed after an appropriate time period.

### 2.5 Fungal infection of plants

Attached wheat leaf infections were performed, as described previously (Rudd et al., 2008) with few modifications. Wheat cultivar Galaxie (Fenaco, Bern, Switzerland) was used for all the plant infections and further details are provided in Kilaru et al. (2015a).

### 2.6 Epi-fluorescent microscopy

Fluorescence microscopy was performed, as described previously (Kilaru et al., 2015b). In brief, the fungal cells were grown in YG media at 18 °C with 200 rpm for 24 h and placed onto a 2% agar cushion and directly observed using a motorized inverted microscope (IX81; Olympus, Hamburg, Germany), equipped with a PlanApo 100x/1.45 Oil TIRF (Olympus, Hamburg, Germany) and a eGFP ET filter-set (470/40 Et Bandpass filter, Beamsplitter T 495 LPXR and 525/50 ET Bandpass filter (Chroma Technology GmbH, Olching, Germany)). The fluorescent tags were excited using a standard mercury burner or a VS-LMS4 Laser Merge System with a 488 nm solid-state laser (75 mW; Visitron Systems, Puchheim, Germany) and imaged in the stream acquisition mode. Average intensity and bleaching behavior of the different green tags were analyzed in movies containing 200 planes captured with 150 ms exposure time and binning 1 using a CoolSNAP HQ2 camera (Photometrics/Roper Scientific, Tucson, USA). All parts of the system were under the control of the software package MetaMorph (Molecular Devices, Wokingham, UK). All statistical analysis was performed using Prism 5.03 (GraphPad Software, La Jolla, USA).

### 2.7 Confocal microscopy of liquid cultures and infected plant tissue

Confocal microscopy of GFPs was done using a Leica SP8 laser scanning confocal microscope (Leica, Wetzlar, Germany), equipped with a HC PL APO CS2 63x/1.40 OIL objective (Leica, Wetzlar, Germany), at 50% argon laser intensity. Acquisition of 200 planes in the stream acquisition mode, using a scan field of 256 × 256 pixels, a scan speed of 700 Hz, a zoom of 1.28 and a resolution of 12 bits, was done in the counting mode of the HyD detector, ranging from 500 nm to 600 nm. Leica LAS AF software was used to analyze the average intensity and bleaching behavior.

For imaging of fungal material in wheat tissue, samples were collected at 14 dpi. Fluorescence was imaged using a Leica SP8 laser scanning confocal microscope (Leica, Wetzlar, Germany) equipped with a HC PL APO CS2 63x/1.40 oil objective (Leica, Wetzlar, Germany) or a HC PL APO CS2 40x/1.30 oil objective (Leica, Wetzlar, Germany). 0.5 cm region was taken from the infected leaf and briefly dipped into Flutec PP11 (F2 Chemicals Ltd., Lea Town, UK) and placed on Carolina observation Gel (Carolina Biological Supply Company, Burlington, USA). To measure the average intensity and bleaching behavior *in planta*, samples were exited using the argon laser at 50% and the counting mode of the HyD detector in a range of 501–530 nm. Images sequences of 150 planes in a scan field of 256 × 256 pixels were acquired, using a scan speed of 600 Hz, zoom 2 and an image bit depth of 12 bit.

Imaging sample represented in Fig. 3C was done using the argon laser at 25% and the emission for the green tags was detected in a range of 497–516 nm with the photon integration mode of the HyD detector with a gain of 133, a scan field of 1024 × 1024 pixels, a scan speed of 400 Hz, a zoom of 2, a line average of 2 and a resolution of 12 bit. Auto-fluorescence of the chloroplasts and plant cell wall was detected using a second HyD detector in a range of 641–678 nm in the photon integration mode with a gain of 10.

### 2.8 Data analysis

The average intensity of the different tags expressed in the fungal cytoplasm was analyzed in the first plane of the generated movies by creating one region of interest (ROI) per cell, covering only a part of the cytoplasm, but excluding the nucleus or vacuoles. A copy of the same ROI was placed next to the cell to analyze the average intensity of the neighboring background. The values of both ROI‘s were transferred to Excel (Microsoft, Redmond, WA, USA) and the values of the neighboring background were subtracted from the values of the cell. All corrected values were copied to Prism 5.03 (GraphPad Software, La Jolla, CA, USA) to perform intensity comparisons.

The bleaching curves were generated by analyzing the average intensity of each plain of the movie as described above. This was done for numerous cells and the mean ± standard deviation of the corrected intensities for each plane was calculated. Curves were drawn in the program Prism 5.03 (GraphPad Software, La Jolla, CA, USA).

To compare the bleaching rate of the various green tags, the measured fluorescent intensity values with time were fitted to a one phase decay model. Decay curves were compared using *F* testing the best fitting decay rates between individual data sets. Fitting and *F* testing are performed in the software Prism 5.03 (GraphPad Software, La Jolla, CA, USA).

## 3 Results and discussion

### 3.1 ZtGFP, encoded by a codon-optimized eGFP

**Fig. 1:**
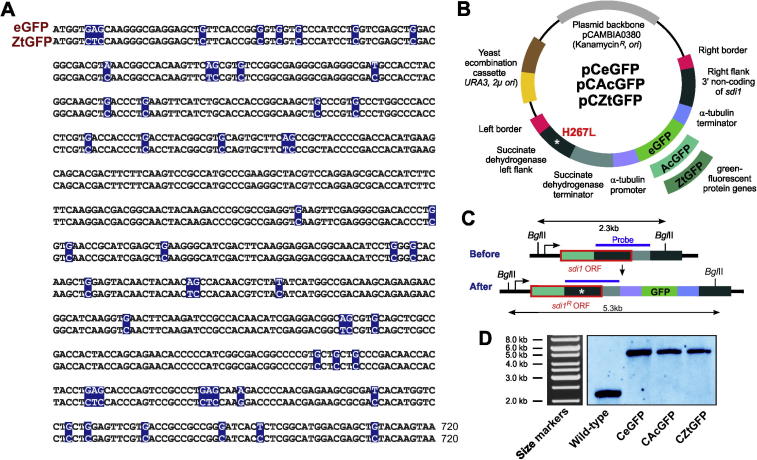
A codon-optimized gene, encoding ZtGFP for use in *Z. tritici.* (A) Nucleotide Fungal Genet. Biol. of the open reading frame of enhanced GFP and a modified GFP, codon-optimized for use in *Z. tritici* (ZtGFP). Nucleotide exchanges are highlighted in blue. Note that these alterations do not modify the translated amino acid sequence. (B) Vector for integration of enhanced GFP from *A. victoria* (eGFP), *A. coerulescens* GFP (AcGFP) and codon-optimized enhanced GFP from *A. victoria* (ZtGFP) into the *sdi1* locus. After integration into the *sdi1* locus, the vector confers carboxin resistance due to a point mutation in the succinate dehydrogenase gene *sdi1*, which changes a histidine to a leucine (H267L). For more details of this integration into the “succinate dehydrogenase locus” see Kilaru et al. (2015a). Left and right border enable *Agrobacterium tumefaciens*-based transformation of *Z. tritici*. (C) Diagram showing the organization of the *sdi1* locus before and after integration of the GFP-encoding vectors. Note that integration of the point mutated *sdi1* left flank (see Fig. 1B; point mutation indicated by asterisk) replaces a part of the *sdi1* open reading frame (*sdi1* ORF) and confers carboxin resistance (*sdi1^R^* ORF). Successful integration of the vector increases the size of a DNA fragment after digestion with the restriction enzyme *Bgl*II and subsequent detection with a labeled DNA probe (blue bar). (D) Southern blot, showing integration of vectors into the *sdi1* locus. After digestion of the genomic DNA with *Bgl*II and subsequent hybridization with a labeled DNA probe a shift in the DNA fragment from 2.3 kb to ∼5.3 kb is detected. The size markers in the corresponding agarose gel are shown to the left.

As a first step toward designing ZtGFP, we received the codon-usage for *Z. tritici*. These data were obtained from the Codon Usage Database (Nakamura et al., 2000). The information was based on 21,315 codons from 34cDNAs in *Z. tritici*. This information was confirmed by analyzing the codon usage from the annotated genome of *Z. tritici* (Goodwin et al. 2011). This analysis revealed that the codons such as GGG (Val), GTA (Val), AGG (Arg), AGA (Arg), AGT (Ser), ATA (Ile), TGT (Cys), TAT (Tyr), TTA (Leu), TCA (Ser), CGG (Arg), CAT (His) and CTA (Leu) were found less frequently in the coding sequences of *Z. tritici.* The amino acid sequence of eGFP was reverse-translated to produce a *Z. tritici* codon-optimized eGFP encoding gene, using the software Sequence Manipulation Suite (Stothard, 2000). The obtained *Z. tritici* codon-optimized eGFP nucleotide sequence contains 54 silent substitutions across the whole length of the sequence (Fig. 1A). We used this information to synthesize commercially the ZtGFP coding-DNA.

### 3.2 Three vectors for targeted ectopic integration of GFP-encoding constructs

To compare the fluorescent brightness and photo-bleaching behavior of AcGFP, eGFP and ZtGFP, we generated three vectors pCAcGFP, pCeGFP and pCZtGFP that allow expression of the GFPs under the *Z. tritici* α-tubulin (*tub2*) promoter (see Schuster et al., 2015) for further details on *tub2;* pCeGFP is described in Kilaru et al., (2015a). We designed all vectors for targeted integration into the genomic *sdi1* locus of *Z. tritici*, by using a mutated downstream stretch of the *sdi1* sequence, carrying a carboxin resistance conferring point mutation (H267L; Fig. 1B, left flank), and a sequence stretch downstream of *sdi1* (Fig. 1B, right flank of *sdi1*). Incorporation by homologous recombination mutates the *sdi1* gene and integrates the GFP constructs into the *sdi1* locus (for details Kilaru et al., 2015a). This yields comparable gene expression levels due to an identical background genomic environment and single integration of each construct, essential for quantitative analysis of fluorescent intensities. All three vectors were built in the *Agrobacterium* binary vector pCAMBIA0380 (CAMBIA, Canberra, Australia) and comprise a “yeast recombination cassette”, consisting of URA3 and 2μ *ori* which enables yeast recombination-based cloning (for more details Kilaru and Steinberg, 2015).

We next transformed all three vectors into *Z. tritici* strain IPO323 (Kema and van Silfhout, 1997) using *A. tumefaciens*-mediated transformation protocol (Zwiers and De Waard, 2001). In order to confirm the single copy integration into the *sdi1* locus, we purified genomic DNA from the transformants and wild-type isolate IPO323, digested all with *Bgl*II and hybridized these to an *sdi1* probe. Indeed, we found a single band at the expected size of ∼5.3 kb in all cases (wild-type locus: ∼2.3 kb; Fig. 1C and D). This confirmed that the GFP constructs were integrated into the *sdi1* locus as single copies resulting in strains IPO323_CAcGFP, IPO323_CeGFP and IPO323_CZtGFP, respectively. In a parallel study, we confirmed that expression of either GFP in the cytoplasm of *Z. tritici* cells did not affect pathogenicity (see Kilaru et al., 2015a). Thus, we have generated three *Z. tritici* strains, expressing non-toxic AcGFP, eGFP and ZtGFP from the same locus and under the same *tub2* promoter. This opened the possibility of comparing their fluorescent brightness and photo-bleaching behaviors.

### 3.3 Fluorescent behavior of AcGFP, eGFP and ZtGFP in epi-fluorescence microscopy of *Z. tritici*

**Fig. 2:**
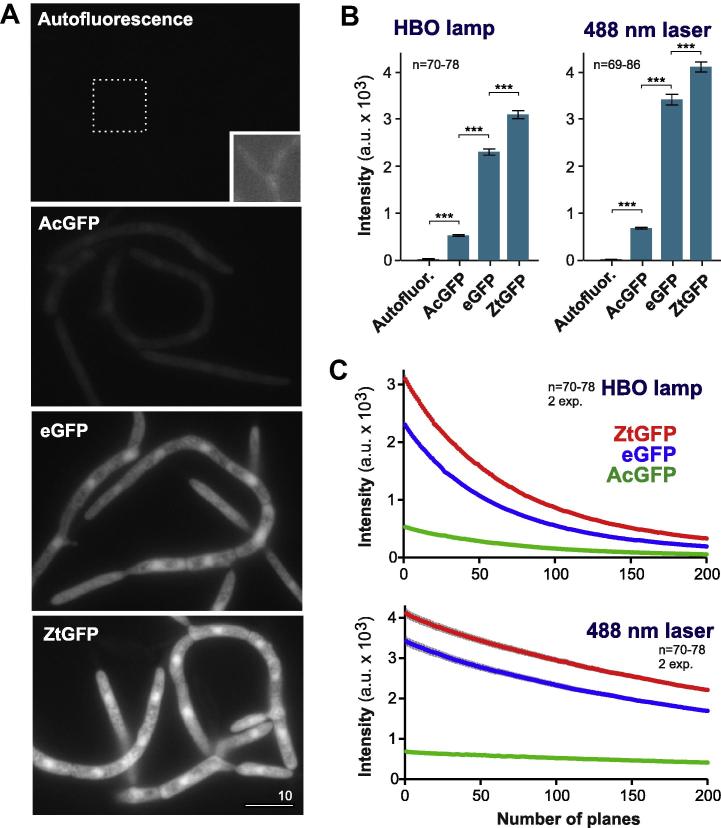
Signal intensity and bleaching behavior of GFP proteins in epi-fluorescence microscopy. (A) Images showing cytoplasmic expression of *A. coerulescens* GFP (AcGFP), enhanced GFP from *A. victoria* (eGFP) and codon-optimized enhanced GFP from *A. victoria* (ZtGFP). Note that *Z. tritici* shows virtually no auto fluorescence (inset shows extreme image processing, showing very low cytoplasmic background). All images were acquired using 150 ms exposure time and binning 1 and identically processed. Bar represents 10 μm. (B) Bar chart showing average intensity of cytoplasmic fluorescence of various GFPs. Autofluor.: background fluorescence without expressing a GFP; AcGFP: cells expressing *A. coerulescens* GFP; eGFP; cells expressing enhanced GFP from *A. victoria*; ZtGFP; cells expressing *Z. tritici* codon-optimized enhanced GFP from *A. victoria.* Mean ± standard error of the mean is shown, sample size *n* is indicated. Triple asterisk indicates significant difference at *P *< 0.0001, Student *t*-test. (C) Graph showing decay of fluorescent signals due to photo-bleaching. AcGFP: cells expressing *A. coerulescens* GFP; eGFP; cells expressing enhanced GFP from *A. victoria*; ZtGFP; cells expressing *Z. tritici* codon-optimized enhanced GFP from *A. victoria.* Each data point is given as mean ± standard error of the mean, sample size *n* is indicated. Note that little variation is found between experiments and that the standard error of the mean is very small.

In a first set of experiments, we investigated one day old liquid cultures of IPO323 and AcGFP, eGFP and ZtGFP expressing strains (IPO323_CAcGFP; IPO323_CeGFP; IPO323_CZtGFP) of *Z. tritici* in conventional epi-fluorescence microscopy, using a HBO mercury short-arc lamp for excitation and identical acquisition settings for all experiments (150 ms exposure time and binning 1). Under these conditions, untransformed IPO323 showed almost negligible fluorescent (Fig. 2A), whereas the GFP-expressing strains showed strong cytoplasmic and nuclear fluorescence (Fig. 2A). That was brightest in ZtGFP-expressing strains, extending eGFP by ∼35% (Fig. 2A and B; significant difference at *P *< 0.0001 is indicated by triple asterisk). We further increased the signal intensity by ∼33% using a 488 nm solid state laser (75 mW) as excitation source and identical acquisition settings for all experiments (150 ms exposure time and binning 1). Again, under these conditions, the brightness of ZtGFP exceeded that of eGFP significantly (Fig. 2B). However, it is worth noting that the measured intensity of ZtGFP differed significantly between experiments. In contrast, AcGFP was relatively faint under all conditions tested (Fig. 2A and B).

We next investigated photo-bleaching behavior in conventional and laser-based epi-fluorescence microscopy. We illuminated single cells, recorded 200 images at 150 ms exposure time using the stream acquisition mode, and measured the average intensity of the cytoplasmic signal in the cell by creating one region of interest (ROI) per cell covering an area of the cytoplasm, but excluding the nucleus and vacuoles. The intensity value of the cell was corrected by the intensity value of the neighboring background. The average fluorescence intensity of all GFPs decreased with time, indicating that all fluorescent proteins undergo photo-bleaching and followed a one-way decay curve (Fig. 2C). In a first set of experiments, we investigated the fluorescent decay with time when a mercury short-arc lamp was used for illumination (HBO lamp). Fitting one-way decay curves and subsequent *F*-testing revealed that AcGFP and eGFP bleached significantly faster than ZtGFP (*P* = 0.0037 and *P* < 0.0001, respectively). Photo-bleaching was much reduced when a 488 nm laser was used for excitation (Fig. 2C, 488 nm laser). Under these conditions, no obvious difference was found for all three GFPs (all *P*-values > 0. 1162). In summary, our analysis revealed that ZtGFP is brighter than eGFP and AcGFP and, at least under HBO illumination, is more photo-stable. Thus, we conclude that the codon-optimized GFP is optimal for fluorescence-based live cell imaging in epi-fluorescence microscopy.

### 3.4 Fluorescent behavior of AcGFP, eGFP and ZtGFP in confocal laser scanning microscopy

**Fig. 3:**
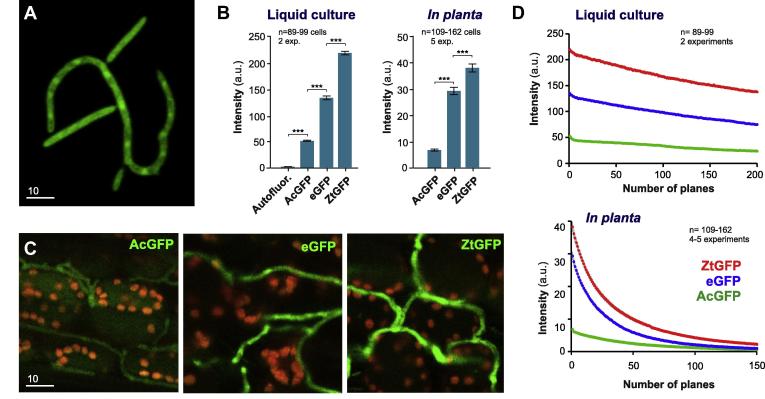
Signal intensity and bleaching behavior of GFP proteins in confocal laser-scanning microscopy. (A) Confocal image of *Z. tritici* cells, grown in liquid culture and expressing ZtGFP. Bar represents 10 μm. (B) Bar chart showing average intensity of cytoplasmic fluorescence of various GFPs, observed with a confocal laser scanning microscope in liquid culture and in infected wheat tissue. Autofluor.: background fluorescence without expressing a GFP; AcGFP: cells expressing *A. coerulescens* GFP; eGFP: cells expressing enhanced GFP from *A. victoria*; ZtGFP: cells expressing codon-optimized enhanced GFP from *A. victoria.* Mean ± standard error of the mean is shown, sample size *n* is indicated. Triple asterisk indicates significant difference at *P *< 0.0001, Student *t*-test. (C) Images of infected wheat tissue at 14 dpi. Hyphal cells express *A. coerulescens* GFP (AcGFP), enhanced GFP from *A. victoria* (eGFP) and codon-optimized enhanced GFP from *A. victoria* (ZtGFP). Auto fluorescence of plant chloroplasts is shown in red. Bar represents 10 μm. (D) Graph showing decay of fluorescent signals due to photo-bleaching in confocal laser scanning microscopy. AcGFP: cells expressing *A. coerulescens* GFP; eGFP: cells expressing enhanced GFP from *A. victoria*; ZtGFP; cells expressing codon-optimized enhanced GFP from *A. victoria.* Each data point is given as mean ± standard error of the mean, sample size *n* is indicated.

Investigation of *Z. tritici* infection stages in wheat tissue requires confocal laser scanning microscopy. We therefore tested signal intensities and photo-bleaching behavior of AcGFP, eGFP and ZtGFP in liquid culture and *in planta* using a Leica TCS SP8L confocal microscope. In liquid culture, ZtGFP shows the strongest cytoplasmic fluorescence, which exceeded that of eGFP by ∼60% (Fig. 3A and B). Again, auto-fluorescence of IPO323 was very minor and AcGFP showed weak fluorescence (Fig. 3B). A similar situation was found *in planta,* 14 days after infection. Again, ZtGFP expression was clearly visible in fungal hyphae (Fig. 3C) and quantitative image analysis revealed that the average signal intensity exceeded that of eGFP significantly (Fig. 3B). No difference in photo-bleaching behavior was observed between the ZtGFP and eGFP (Fig. 3D, *P* = 0.1503; *F*-test after fitting one-way decay curves) when cells were grown in liquid culture. However, eGFP fluorescence decayed significantly faster than ZtGFP when cells were observed *in planta* (Fig. 3D, *P* < 0.0001; *F*-test after fitting one-way decay curves). These results confirm the previous outcome for HBP illumination and show that the ZtGFP is also optimal for confocal laser scanning microscopy of *Z. tritici*, both in liquid culture and in infected plant tissue.

## 4 Conclusion

In this study, we generated a codon-optimized GFP for use in *Z. tritici* (ZtGFP). We introduced 54 silent mutations into the eGFP sequence in order to optimize its codons for expression in *Z. tritici* and compared fluorescent intensity and photo-bleaching behavior of cytoplasmic ZtGFP to eGFP and AcGFP in several microscopic systems. Overall, AcGFP performed very poorly and is not recommended for use in *Z. tritici*. eGFP and ZtGFP provide bright signals and can be used to observe the fungus inside infected plant tissue. However, ZtGFP is superior, as it is significantly brighter and significantly more photo-stable when expressed in the cytoplasm of *Z. tritici*. The difference was most obvious in confocal laser scanning microscopy, which is the method of choice for analyzing host-pathogen interaction. We conclude that ZtGFP is the fluorescent protein of choice to investigate cellular protein dynamics or host-pathogen interaction in the wheat pathogen *Z. tritici*.

## Figures and Tables

**Table 1:** Primers used in this study.

Primer name	Direction	Sequence (5′ to 3′)^a^
SK-Sep-10	Sense	*TGGCAGGATATATTGTGGTGTAAACAAATT*GACCTTCCACATCTACCGATGG
SK-Sep-13	Antisense	CTTCCGTCGATTTCGAGACAGC
SK-Sep-14	Sense	*CATTTGCGGCTGTCTCGAAATCGACGGAAG*GCAGTCGACGCCAGATGATGG
SK-Sep-15	Antisense	*GGTGAACAGCTCCTCGCCCTTGCTCACCAT*GGCGATGGTGGTATGCGGATG
SK-Sep-79	Sense	*CATCACTCACATCCGCATACCACCATCGCC*ATGGTGAGCAAGGGCGCCGAG
SK-Sep-80	Antisense	*CCACAAGATCCTGTCCTCGTCCGTCGTCGC*TCACTTGTACAGCTCATCCATGC
SK-Sep-101	Sense	*CATCACTCACATCCGCATACCACCATCGCC*ATGGTCTCCAAGGGCGAGGAG
SK-Sep-102	Antisense	*CCACAAGATCCTGTCCTCGTCCGTCGTCGC*TTACTTGTAGAGCTCGTCCATGC

a Italics indicate part of the primer that is complementary with another DNA fragment, to be ligated by homologous recombination in *S. cerevisiae*.
